# Research trends on autoimmune liver diseases and gut microbiota (1989–2025): a bibliometric and visualization analysis based on the web of science core collection database

**DOI:** 10.3389/fimmu.2026.1785064

**Published:** 2026-04-29

**Authors:** Ying Wan, Mengyao Zheng, Wen Fu, Huiling Zhu, Yuting Li, Huiying Lin, Jie Chen, Jianwei Li, Qiulan Mo, Wenlin Tai, Jinhui Yang

**Affiliations:** 1 Department of Gastroenterology, The Second Affiliated Hospital of Kunming Medical University, Kunming, China; 2 Laboratory Science Department, The Second Affiliated Hospital of Kunming Medical University, Kunming, China

**Keywords:** autoimmune hepatitis, autoimmune liver diseases, bibliometric, gut microbiota, primary biliary cholangitis, primary sclerosingcholangitis, scopus

## Abstract

Autoimmune liver diseases (AILD) encompass autoimmune hepatitis (AIH), primary biliary cholangitis (PBC), primary sclerosing cholangitis (PSC), and IgG4-related sclerosing cholangitis (IgG4-SC), with both incidence and prevalence showing an upward trend in recent years. Increasing evidence indicates that the onset and progression of AILD are inextricably linked to the gut microbiota. So far, no comprehensive and objective bibliometric study on AILD and gut microbiota has been conducted. This study retrieved literature from the Web of Science Core Collection (WoSCC) and Scopus database up to October 13, 2025, for analysis and validation respectively. VOSviewer, CiteSpace, R, Bibliometrix, SciExplorer, and WPS Office analysis tools were employed to systematically evaluate research trends, hot topics, and knowledge structures in the field. Our analysis indicates a growing research trend in AILD-gut microbiota interactions, involving 58 countries, 981 institutions, and 3,528 authors. Influential journals in this field include *Frontiers in Immunology*, *GUT*, and *World Journal of Gastroenterology*. Gershwin M. Eric stands as an authoritative author in this domain. Hot topics include “Mendelian randomization”,”primary sclerosing cholangitis”, “primary biliary cholangitis”, “bile acids”, and the “gut-liver axis”. Exploring therapeutic targets in AILD patients through the gut microbiome and its metabolites may emerge as a key future research direction. In summary, this study employed bibliometric methods to evaluate the application and development of gut microbiota in the field of AILD. Research in this area has experienced rapid growth in recent years, progressively focusing on the mechanisms of the gut-liver axis and genetics. Future efforts should further explore the potential of gut microbiota modulation in AILD treatment.

## 1 Introduction

Autoimmune liver disease (AILD) is a group of chronic liver diseases caused by immune system abnormalities. It is characterized by chronic inflammation of the liver, damage to hepatocytes or bile duct structures (1). AILD encompasses autoimmune hepatitis (AIH), primary biliary cholangitis (PBC), primary sclerosing cholangitis (PSC), and IgG4-related sclerosing cholangitis (IgG4-SC). The latest research indicates that the global prevalence of AIH is approximately 15.65 per 100,000 people, while PBC has a prevalence of about 14.60 per 100,000 people. The prevalence rates for PSC and IgG4-SC are lower than those of the first two conditions (2, 3). Although AILD is a rare disease, both its incidence and prevalence have shown an upward trend in recent years. As the disease progresses, it may lead to complications such as liver fibrosis, cirrhosis, and end-stage liver disease (including the need for liver transplantation), significantly impacting patient survival rates and quality of life. Despite extensive research on the complex interactions between genetic susceptibility, immune responses, and environmental factors (4, 5), the pathogenesis of AILD remains incompletely understood. Existing findings cannot fully explain the heterogeneity observed among patients in terms of clinical presentation, disease progression, treatment response, and prognosis.

In recent years, with the development of concepts surrounding the gut microbiota and the gut-liver axis, the gut microbiota has been proposed as a crucial bridge connecting the environment, metabolism, immunity, and hepatic pathology. This offers new perspectives for exploring the disease mechanisms of AILD and innovating treatment strategies. Most studies report significantly reduced α-diversity in the gut microbiota of AILD patients, accompanied by increased abundance of harmful bacteria and decreased abundance of protective commensal bacteria and short-chain fatty acid-producing functional bacteria (6–12). Mechanistic research suggests that disruption of the intestinal barrier and release of microbial metabolites can enter the liver via the enterohepatic circulation, activating hepatic immune responses and promoting inflammatory and fibrotic processes (13, 14). Additionally, studies employing statistical genetic methods such as Mendelian randomization (MR) to investigate potential causal relationships among gut microbiota, immune cells, and AILD have recently emerged, providing preliminary evidence for the microbiota as a causal factor (rather than merely a correlated one) (15, 16). However, most current studies on AILD and gut microbiota remain cross-sectional or small-sample cohort studies, often focusing on single disease models and single geographic/population settings, lacking comprehensive and longitudinal research.

Bibliometrics is a scientific research method for quantitatively analyzing literature. By examining factors such as publication volume, author collaboration, and keyword co-occurrence, it reveals development trends and hot topics within academic research fields. In recent years, the use of bibliometric analysis in studying various diseases has grown steadily. However, systematic bibliometric analysis remains scarce in the field of AILD and gut microbiota. Given this background and gap, this study adopts a global perspective, systematically reviews research in this domain using publicly available literature published between 1989 and 2025, and combines quantitative analysis with visualization network. This provides a reference for future research and applications.

## 2 Materials and methods

### 2.1 Data source and search strategy

Web of Science (WoS) is the world’s leading academic database, providing multidisciplinary, high-quality scholarly resources widely used in literature retrieval, citation analysis, and research evaluation (17). In this study, we selected literature from the Web of Science Core Collection (WoSCC) for bibliometric analysis. We employed the following search strategy for retrieval:TS=(“Autoimmune liver disease” OR “Primary biliary cholangitis” OR “Primary biliary cirrhosis” OR “Autoimmune hepatitis” OR “Primary sclerosing cholangitis” OR “IgG4-related sclerosing cholangitis” OR “AILD” OR “PBC” OR “PSC” OR “AIH” OR “IgG4-SC”) AND TS=(“gut” OR “intestinal” OR “gastrointestinal”) AND TS=(“microbiota” OR “microbiome” OR “flora” OR “microflora” OR “bacteria” OR “microbial” OR “microbe” OR “microbes” OR “microorganism” OR “microorganisms”). The search period spans from the database’s inception to October 13, 2025. Article types were restricted to “article” and “review” with language limited to English. The search yielded a total of 651 articles. The retrieval results are exported as plain text files and saved in.txt format.

### 2.2 Data analysis

Analytical tools VOSviewer (version 1.6.20), CiteSpace (version 6.4.R1), R-Bibliometrix (version 5.0), R 4.5.1, Open Scientometrics Data Analysis and Visualization Platform (SciExplorer) and WPS Office 2025 were employed to analyze all data retrieved from WOSCC, generating visualizations for both quantitative and qualitative analysis. VOSviewer (Version 1.6.20) was used to visualize co-occurrence networks for journals, countries, institutions, authors, citations, references, and keywords. Each node in the co-occurrence networks represents a publication, country, institution, author, or keyword, grouped according to their respective type. CiteSpace (version 6.4.R1) was employed to analyze references and keywords with the strongest citation bursts, generate journal overlay maps, perform keyword cluster analysis and keyword timeline view. R-Bibliometrix (version 5.0) was employed to obtain general information on all publications, including publication volumes by year, journal, country, institution, and author. It also yielded keywords distribution over time and most cited publications. We further leveraged the powerful capabilities of R 4.5.1 to enhance the visual representation of research findings. SciExplorer was utilized to generate collaboration chord diagrams and collaboration map for countries/regions. Finally, WPS Office 2025 was employed to generate a keyword word clouds.

### 2.3 Validation analysis

To assess the robustness of the bibliometric findings derived from the WoS database, an independent validation analysis was conducted using the Scopus database. The same search strategy, language restriction (English), article types (article and review), and time span were applied to ensure comparability between the two datasets. Key bibliometric indicators, including annual publication trends, high-frequency keywords, leading contributing journals, leading contributing countries/regions and leading contributing authors, were compared between the WoS and Scopus to evaluate the consistency of the main findings.

## 3 Results

### 3.1 Basic information

**Figure 1:**
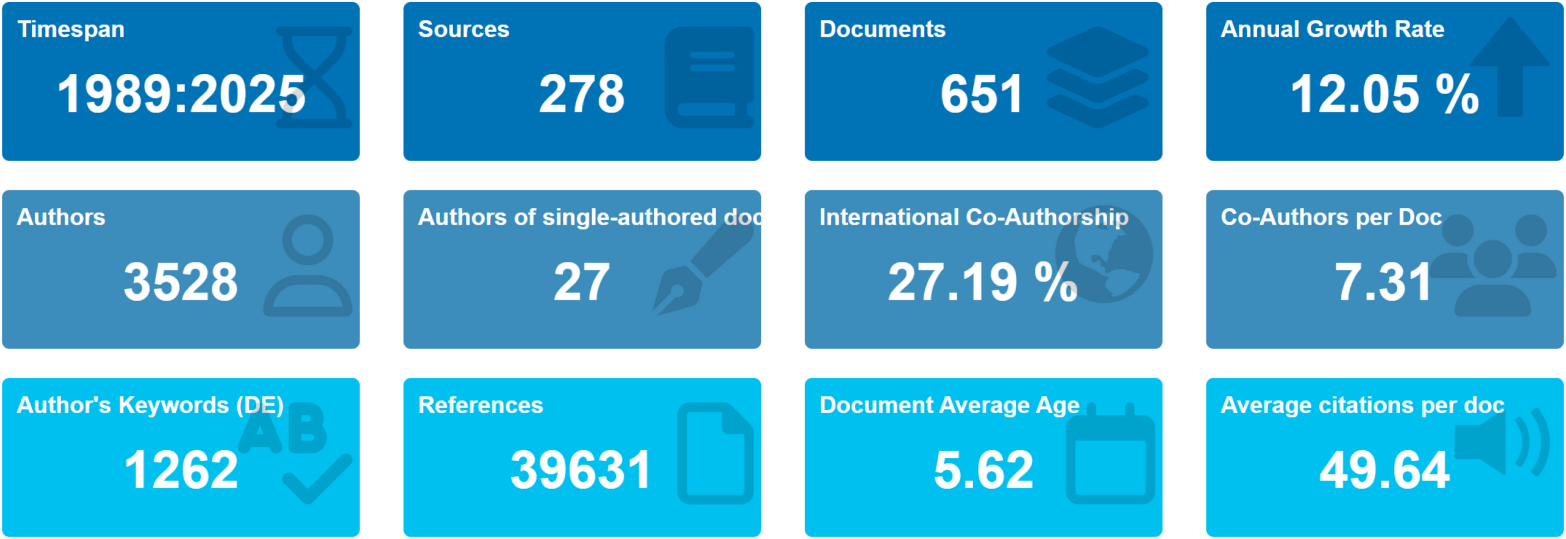
Basic information of the literature.

As shown in **Figure 1**, the 651 documents included in this study were authored by 3,528 researchers from 981 institutions across 58 countries and regions. They cited 39,631 documents and were published in 278 journals, with an annual growth rate of 12.05%. Among these, 27 documents were single-authored, and international collaboration accounted for 27.19%. Each paper had an average of 7.31 co-authors, with an average publication age of 5.62 years and an average citation count of 49.64 per paper. The field generated 1,262 keywords, reflecting its high output, strong citation impact, and robust international collaboration trends.

### 3.2 Annual publications of the literature included

**Figure 2:**
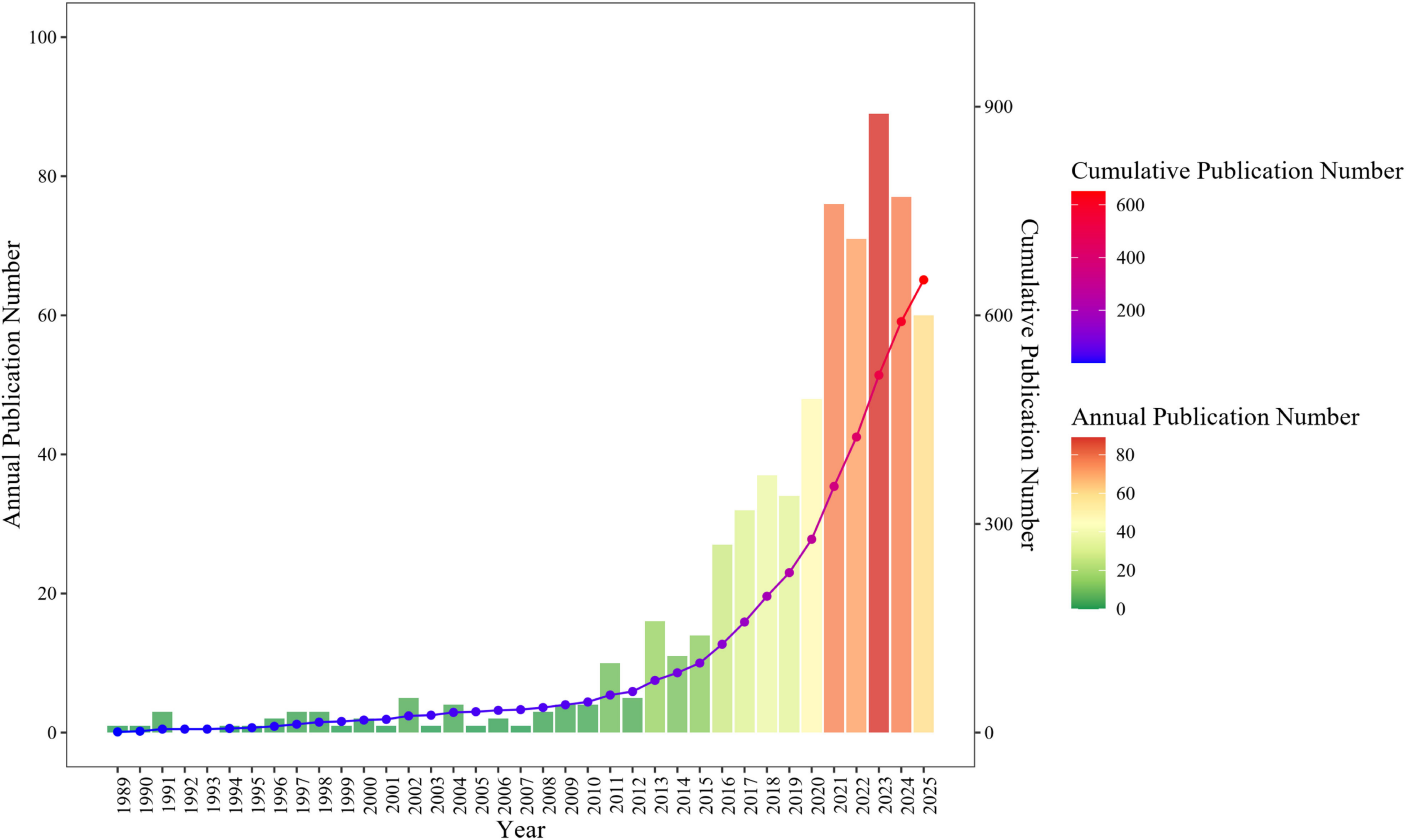
Annual publication volume and cumulative publication volume of AILD and gut microbiota research literature from 1989 to 2025. The bar chart represents the annual publication volume of articles, while the line chart shows the cumulative publication volume of articles.

We compiled a total of 651 publications on AILD and gut microbiota from the WoSCC database, including 343 articles and 308 reviews. We created a dual-axis chart (line graph and bar chart) to illustrate the evolution of publications in this field from 1989 to 2025 (**Figure 2**). The annual publication volume in AILD and gut microbiota research shows a significant overall upward trend. Based on annual publication growth rates, its development can be divided into three phases: the Emergence Phase (1989–2009), characterized by consistently low annual publication volumes (0–5 papers/year), low annual growth rates, and limited fluctuations; Growth Phase (2010–2018): Annual publications increased from 4 to 37, reflecting significantly heightened research activity; Explosion Phase (2019–2025): Annual publications experienced exponential growth, maintaining consistently high output levels. The cumulative publication curve also exhibits sustained upward trajectory.

### 3.3 Journals and discipline analysis

**Figure 3:**
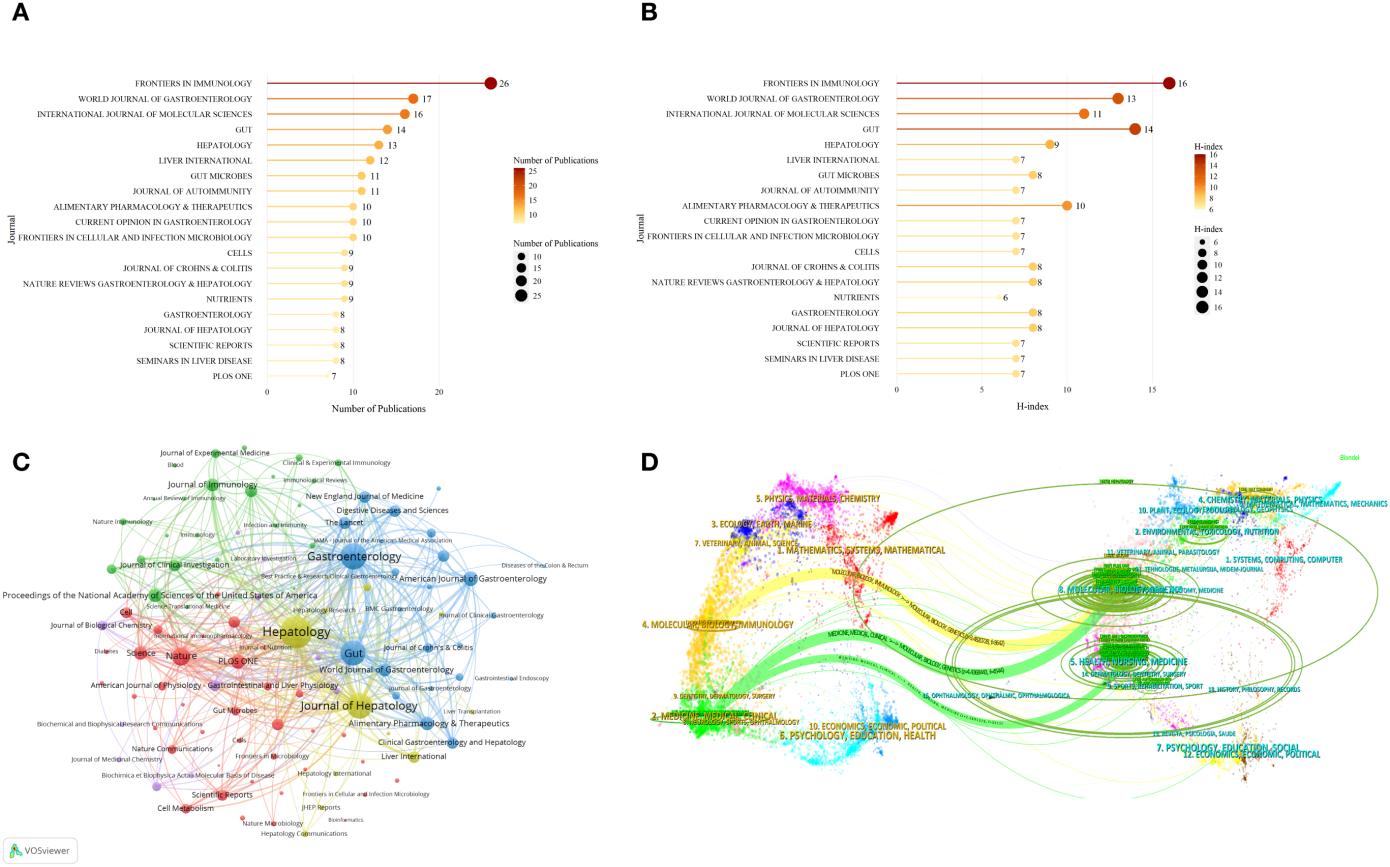
The most productive and influential journals in the field of AILD and gut microbiota research. **(A)** Top 20 journals ranked by publication volume. Darker colors and larger circles indicate higher journal publication volumes. The number adjacent to each circle indicates the exact publication volume of the corresponding journal. **(B)** Top 20 journals ranked by H-index. Darker colors and larger circles indicate higher journal H-index values. The number adjacent to each circle indicates the exact H-index of the corresponding journal. **(C)** Journals co-citation network. Nodes of different colors represent the journals in different clusters. The size of the nodes indicates total link strength, and the thickness of the lines represents the link strength between two journals. **(D)** The dual-map overlay of journals. Citing journals are displayed on the left, and cited journals are on the right. Colored lines indicate the citation relationships between them.

**Figures 3A, B** show the top 20 journals ranked by publication volume and H-index in the fields of AILD and gut microbiota research from 1989 to 2025. The H-index is a metric that measures both the productivity and citation impact of a scholar’s or journal’s published articles (18). These journals have collectively published 225 articles, accounting for 34.56% of the total publications. *Frontiers in Immunology* leads with the highest publication count (26 articles) and H-index (16), reflecting its significant academic influence, both in terms of high output and extensive citation. Other journals, such as *Gut* and *World Journal of Gastroenterology*, demonstrate their importance in the field through their higher H-index values. Although some journals, like *Seminars in Liver Disease* and *Plos One*, have a lower publication volume, their higher H-index indicates their strong citation rates and substantial academic impact.

**Figure 3C** presents the journal co-citation network for AILD and gut microbiota research, visualized using VOSviewer. The core journals with the highest total link strength are *Hepatology*, *Gastroenterology*, *Gut*, and *Journal of Hepatology*, forming a key academic network that dominates the field. These journals exhibit strong co-citation relationships with immunology journals like *Journal of Immunology*, gut microbiota-related journals such as *Gut Microbes*, and interdisciplinary journals like *Nature* and *Science*. Overall, the network in the figure reflects the increasing interdisciplinary collaboration and mutual influence across multiple research areas.

As shown in **Figure 3D**, labels correspond to the disciplines covered by the journals, while colored paths represent citation relationships. Yellow paths indicate that research published in Molecular/Biology/Genetics journals is frequently cited by Molecular/Biology/Immunology journals. Green paths indicate that research from Molecular/Biology/Genetics and Health/Nursing/Medicine journals is frequently cited by Medicine/Medical/Clinical journals.

### 3.4 Analysis of countries/regions

**Figure 4:**
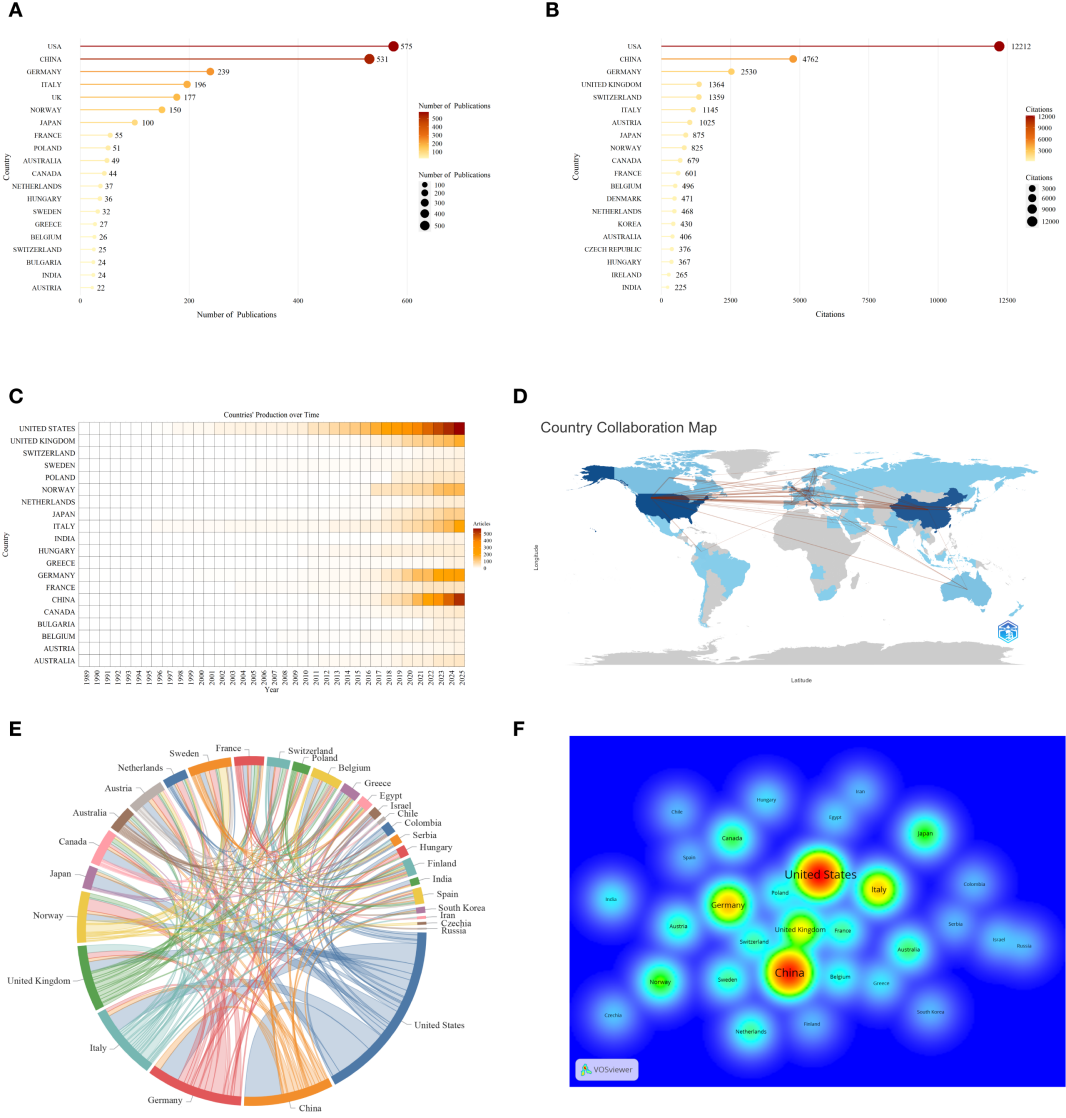
Countries/regions analysis of research on AILD and gut microbiota. **(A)** The top 20 countries with the highest productivity. Darker colors and larger circles indicate higher country publication volumes. The number adjacent to each circle indicates the exact publication volume of the corresponding country. **(B)** The top 20 countries with the most citations. Darker colors and larger circles indicate higher country citations. The number adjacent to each circle indicates the exact citation of the corresponding country.**(C)** Heatmap of countries^,/^regions,production over time. Darker colors indicate higher annual publication volumes. **(D)** Countries/regions collaboration map. The lines between countries/regions represent their collaboration, and the thickness of the lines represents the number of collaborations. **(E)** Countries/regions collaboration chord chart. The lines between countries/regions represent their collaboration, and the thickness of the lines represents the number of collaborations. **(F)** Density visualization of countries/regions collaboration. Warmer colors indicate higher collaboration intensity.

The global landscape of academic output reveals pronounced national disparities (as shown in **Figures 4A, B**). The United States leads by a wide margin with 575 publications, followed by China (531), Germany (239), and Italy (196). In terms of citations, the United States also leads in academic influence (12,212 citations), followed by China (4,762 citations) and Germany (2,530 citations). Despite lower publication volumes, countries like Switzerland (25 papers) and Austria (22 papers) achieve high citation rates globally (1,359 citations and 1,025 citations, respectively) due to the high quality of their research. The annual publication volume heatmap (**Figure 4C**) reveals pronounced “recent concentration” and “head clustering” characteristics in this field. Activity remained sparse across nations before 2015, rapidly intensified starting in 2016, and peaked between 2020 to 2024. The United States has long led in publication volume. China surged starting in 2018 and has driven alongside the U.S. in recent years, while the United Kingdom, Germany, and Italy consistently occupy the second tier.

**Figures 4D–F**, utilizing R-bibliometrix and VOSviewer, illustrate that global research collaboration in this field exhibits a “dual-core” and “multipolar” structure. Primary collaboration corridors span the Atlantic and Pacific Rim, with extensive transcontinental research ties between Europe/America and Asia. The United States and China exhibit particularly strong collaborative clustering effects, while Germany, the United Kingdom, Italy, and others form secondary cooperation hubs. In contrast, South America and Africa show relatively low participation in these collaborations.

### 3.5 Analysis of institutions

**Figure 5:**
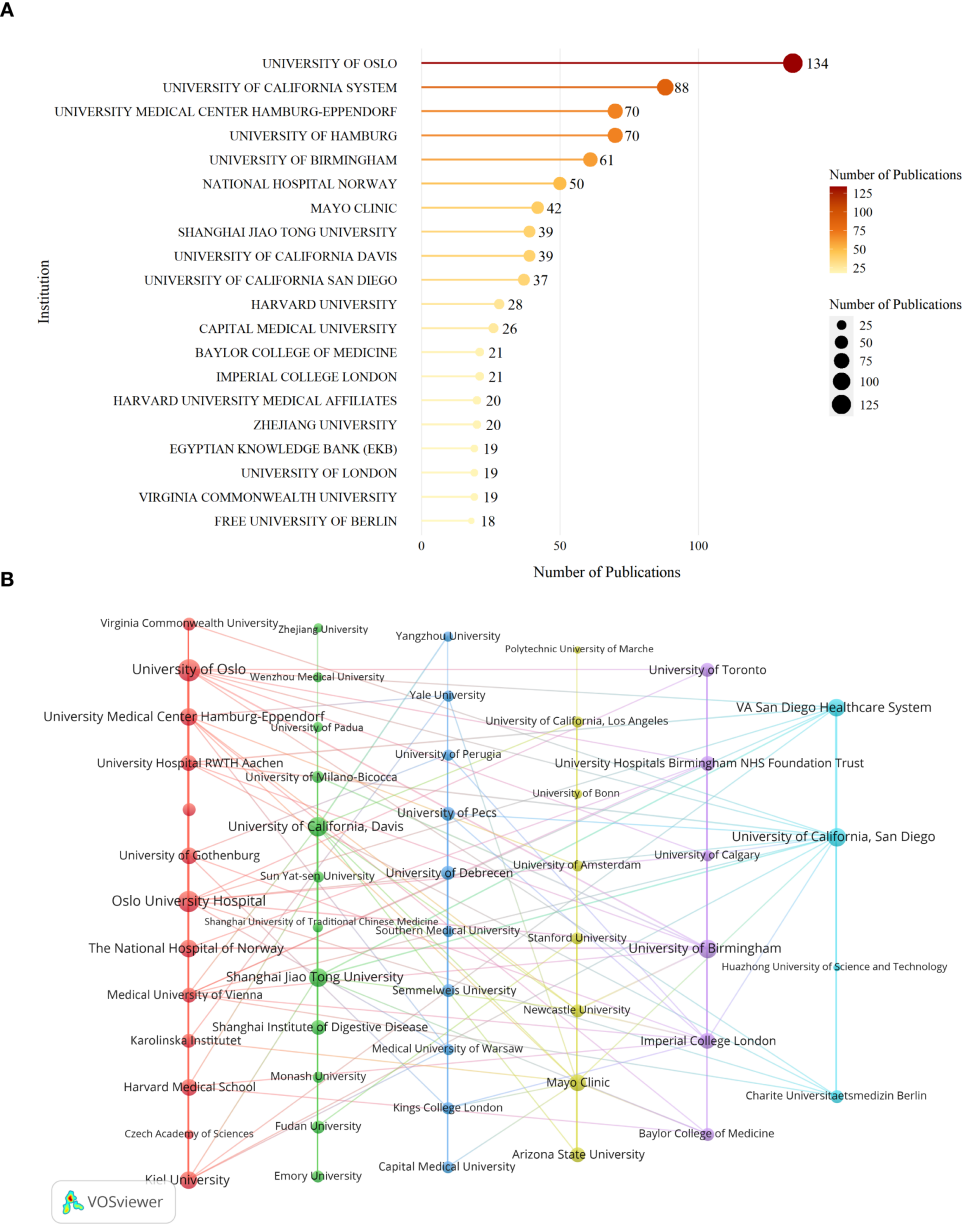
Institutions analysis of research on AILD and gut microbiota. **(A)** The top 20 institutions with the highest productivity. Darker colors and larger circles indicate higher institution publication volumes. The number adjacent to each circle indicates the exact publication volume of the corresponding institution. **(B)** Institutions co-citation network. Nodes of different colors represent the institutions in different clusters. The size of the nodes indicates total link strength, and the thickness of the lines represents the link strength between two institutions.

**Figure 5A** displays the top 20 institutions by publication volume in the field of AILD and gut microbiota research. Among these, 8 are located in the United States, 3 each in Germany, the United Kingdom, and China, 2 in Norway, and 1 in Egypt. The institution with the highest number of publications is University of Oslo (134 papers), followed by University of California System (88 papers), University Medical Center Hamburg-Eppendorf (70 papers), and University of Hamburg (70 papers). **Figure 5B** indicates that the University of Oslo exhibits the strongest collaborative ties with other institutions, with a total link strength of 62. Oslo University Hospital follows with a total link strength of 52. University of Oslo and Oslo University Hospital (19 links) rank first in collaboration frequency, while the University of California, San Diego and VA San Diego Healthcare System (14 links) rank second.

### 3.6 Analysis of authors

**Figure 6:**
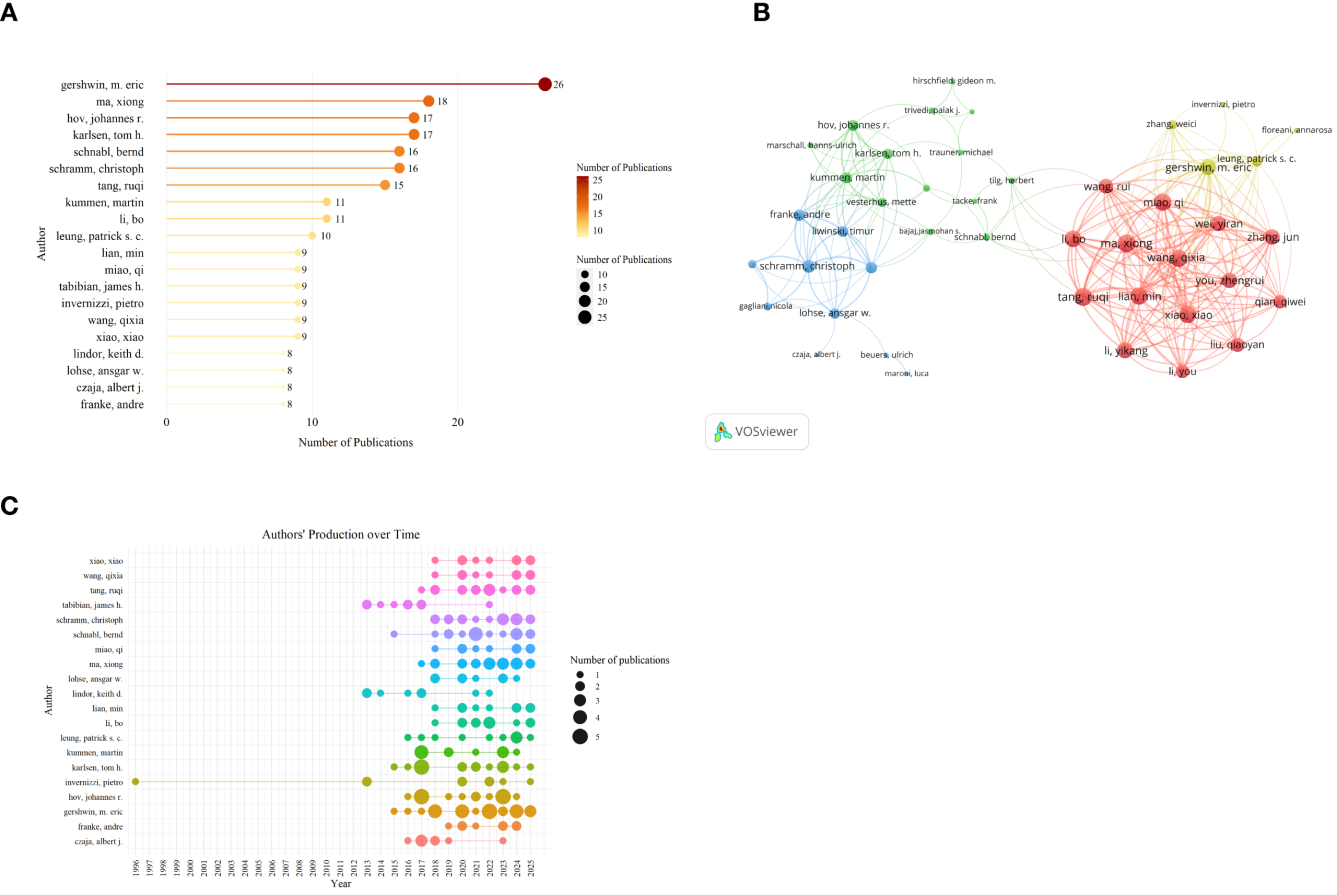
Authors analysis of research on AILD and gut microbiota. **(A)** The top 20 authors with the highest productivity. Darker colors and larger circles indicate higher author publication volumes. The number adjacent to each circle indicates the exact publication volume of the corresponding author. **(B)** Authors co-citation network. Nodes of different colors represent the authors in different clusters. The size of the nodes indicates total link strength, and the thickness of the lines represents the link strength between two authors. **(C)** Authors,production over time. Larger circles indicate higher annual publication volumes.

Research in this field involves 3,528 authors. The top 20 authors by publication volume collectively authored 243 papers, accounting for 37.33% of all published works (**Figure 6A**). Gershwin M. Eric leads with 26 papers; followed by Ma Xiong (18 papers), Hov Johannes R. and Karlsen Tom H. (17 papers each), with most others publishing between 8–11 papers. In the collaboration network (**Figure 6B**), Ma Xiong exhibits the strongest collaborative ties with others, with a total link strength of 122. The collaborative network is primarily divided into three major modules: a high-density Chinese team centered around Ma Xiong, Tang Ruqi, Lian Min, and others; a stable European team anchored by Schramm Christoph, Franke Andre, Kummen Martin, Hov Johannes R., and others; and cross-group bridges anchored by Schnabl Bernd, Tilg Herbert and others.

**Figure 6C** illustrates the annual publication trends of the top 20 authors. The sustained high productivity of core authors primarily began after 2016, though individual senior scholars like Invernizzi Pietro produced early works in 1996 and 2013. New-generation authors (e.g., Xiao Xiao, Wang Qixia) gradually joined the scene after 2018 and maintained stable output. This “senior anchors + new generation relay” structure, coupled with cross-group bridging, jointly drives the rapid diffusion of knowledge.

### 3.7 Analysis of cited literature and references

**Figure 7:**
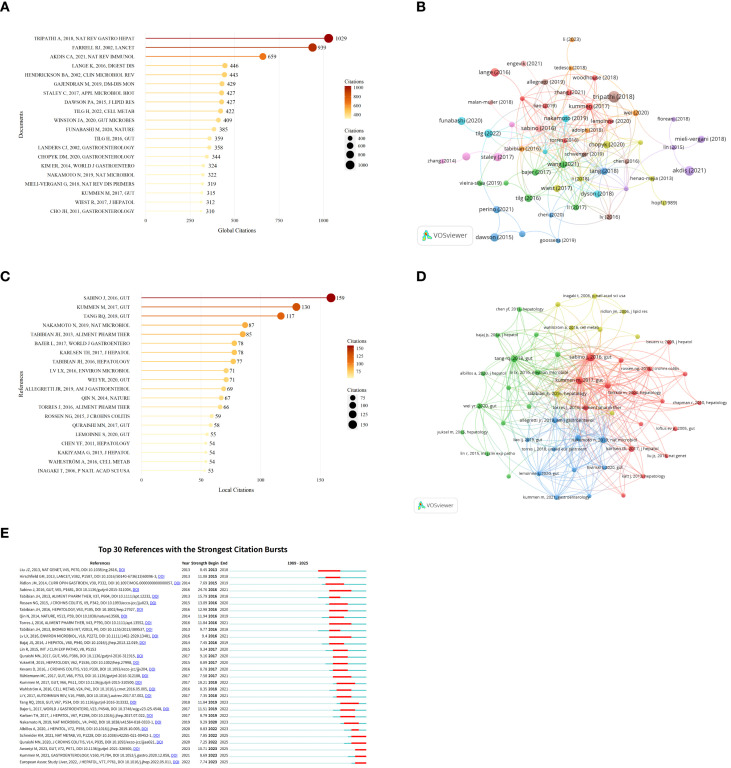
Cited literature and references analysis research on AILD and gut microbiota. **(A)** Top 20 most global cited documents. Darker colors and larger circles indicate higher global citation counts. The number adjacent to each circle indicates the exact global citation count of the corresponding literature. **(B)** Global cited documents co-citation network. Nodes of different colors represent the literatures in different clusters. The size of the nodes indicates the total citation frequency of the literatures, and the thickness of the lines represents the co-citation strength between two literatures. **(C)** Top 20 most local cited documents. Darker colors and larger circles indicate higher local citation counts. The number adjacent to each circle indicates the exact local citation count of the corresponding literature. **(D)** Local cited documents co-citation network. Nodes of different colors represent the literatures in different clusters. The size of the nodes indicates the total citation frequency of the literatures, and the thickness of the lines represents the co-citation strength between two literatures. **(E)** Top 30 references with the strongest citation bursts. The red segment indicates the time span of the reference’s citation burst.

Among the 651 articles included in this study, the paper titled “The gut–liver axis and the intersection with the microbiome” (14) by Tripathi A et al., published in Nature Reviews Gastroenterology & Hepatology, received the highest number of global citations (**Figures 7A, B**). This review examines gut-liver communication mechanisms across various liver diseases, explores molecular, genetic, and microbiome interactions, highlights microbiome-based therapeutic approaches, and envisions the prospect of using the microbiome to stage liver disease, predict outcomes of drug interventions, dietary interventions, and other therapeutic measures at both population and individual levels. It also outlines experimental designs within this research field. **Table 1** lists information on the top 10 global most cited works in this research domain.

**Table 1:** The top 10 global most cited literature in research on AILD and gut microbiota.

Rank	Title	First author	Journal	Year	Citations
1	The gut–liver axis and the intersection with the microbiome (14)	Tripathi A	Nat Rev Gastro Hepat	2018	1029
2	Ulcerative colitis (68)	Farrell Rj	Lancet	2002	939
3	Does the epithelial barrier hypothesis explain the increase in allergy, autoimmunity and other chronic conditions? (69)	Akdis Ca	Nat Rev Immunol	2021	659
4	Effects of Antibiotics on Gut Microbiota (70)	Lange K	Digest Dis	2016	446
5	Clinical Aspects and Pathophysiology of Inflammatory Bowel Disease (71)	Hendrickson Ba	Clin Microbiol Rev	2002	443
6	A comprehensive review and update on ulcerative colitis (72)	Gajendran M	Dm-Dis Mon	2019	429
7	Interaction of gut microbiota with bile acid metabolism and its influence on disease states (44)	Staley C	Appl Microbiol Biot	2017	427
8	Intestinal transport and metabolism of bile acids (73)	Dawson Pa	J Lipid Res	2015	427
9	Gut-liver axis: Pathophysiological concepts and clinical implications (74)	Tilg H	Cell Metab	2022	422
10	Diversification of host bile acids by members of the gut microbiota (75)	Winston Ja	Gut Microbes	2020	409

Among all references, the article “Primary sclerosing cholangitis is characterized by intestinal dysbiosis independent from IBD” (19) by Sabino J et al., published in *Gut*, received the highest number of local citations (**Figures 7C, D**). This paper first proposed PSC-associated fecal microbial imbalance characteristics independent of IBD, suggesting that the gut microbiota may be a contributing factor in the pathogenesis of PSC. **Table 2** lists information on the top 10 local most frequently cited references in this research field.

**Table 2:** The top 10 local most cited references in research on AILD and gut microbiota.

Rank	Title	First author	Journal	Year	Citations
1	Primary sclerosing cholangitis is characterized by intestinal dysbiosis independent from IBD (19)	Sabino J	Gut	2016	159
2	The gut microbial profile in patients with primary sclerosing cholangitis is distinct from patients with ulcerative colitis without biliary disease and healthy controls (76)	Kummen M	Gut	2017	130
3	Gut microbial profile is altered in primary biliary cholangitis and partially restored after UDCA therapy (35)	Tang Rq	Gut	2018	117
4	Gut pathobionts underlie intestinal barrier dysfunction and liver T helper 17 cell immune response in primary sclerosing cholangitis (77)	Nakamoto N	Nat Microbiol	2019	87
5	Randomized clinical trial: vancomycin or metronidazole in patients with primary sclerosing cholangitis - a pilot study (78)	Tabibian Jh	Aliment Pharm Ther	2013	85
6	Distinct gut microbiota profiles in patients with primary sclerosing cholangitis and ulcerative colitis (79)	Bajer L	World J Gastroentero	2017	78
7	Primary sclerosing cholangitis - a comprehensive review (80)	Karlsen Th	J Hepatol	2017	78
8	Absence of the intestinal microbiota exacerbates hepatobiliary disease in a murine model of primary sclerosing cholangitis (81)	Tabibian Jh	Hepatology	2016	77
9	Alterations and correlations of the gut microbiome, metabolism and immunity in patients with primary biliary cirrhosis (82)	Lv Lx	Environ Microbiol	2016	71
10	Alterations of gut microbiome in autoimmune hepatitis (10)	Wei Yr	Gut	2020	71

**Figure 7E** uses CiteSpace to display the top 30 most cited references in this field, with red lines indicating periods of significant citation surges. The earliest citation surge was observed in 2013, with the paper titled “Dense genotyping of immune-related disease regions identifies nine new risk loci for primary sclerosing cholangitis” (20). The strongest surge (intensity = 24.76) occurred in 2016 with Sabino J et al.’s *Gut* publication “Primary sclerosing cholangitis is characterized by intestinal dysbiosis independent from IBD” (19) persisting through 2021. Results indicate the highest citation surge volume occurred between 2016 -2017, reflecting peak research activity during these years. Currently, five articles are still remaining in the citation surge phase, representing intervenable targets and clinical application directions.

### 3.8 Keywords and hotspots analysis

**Figure 8:**
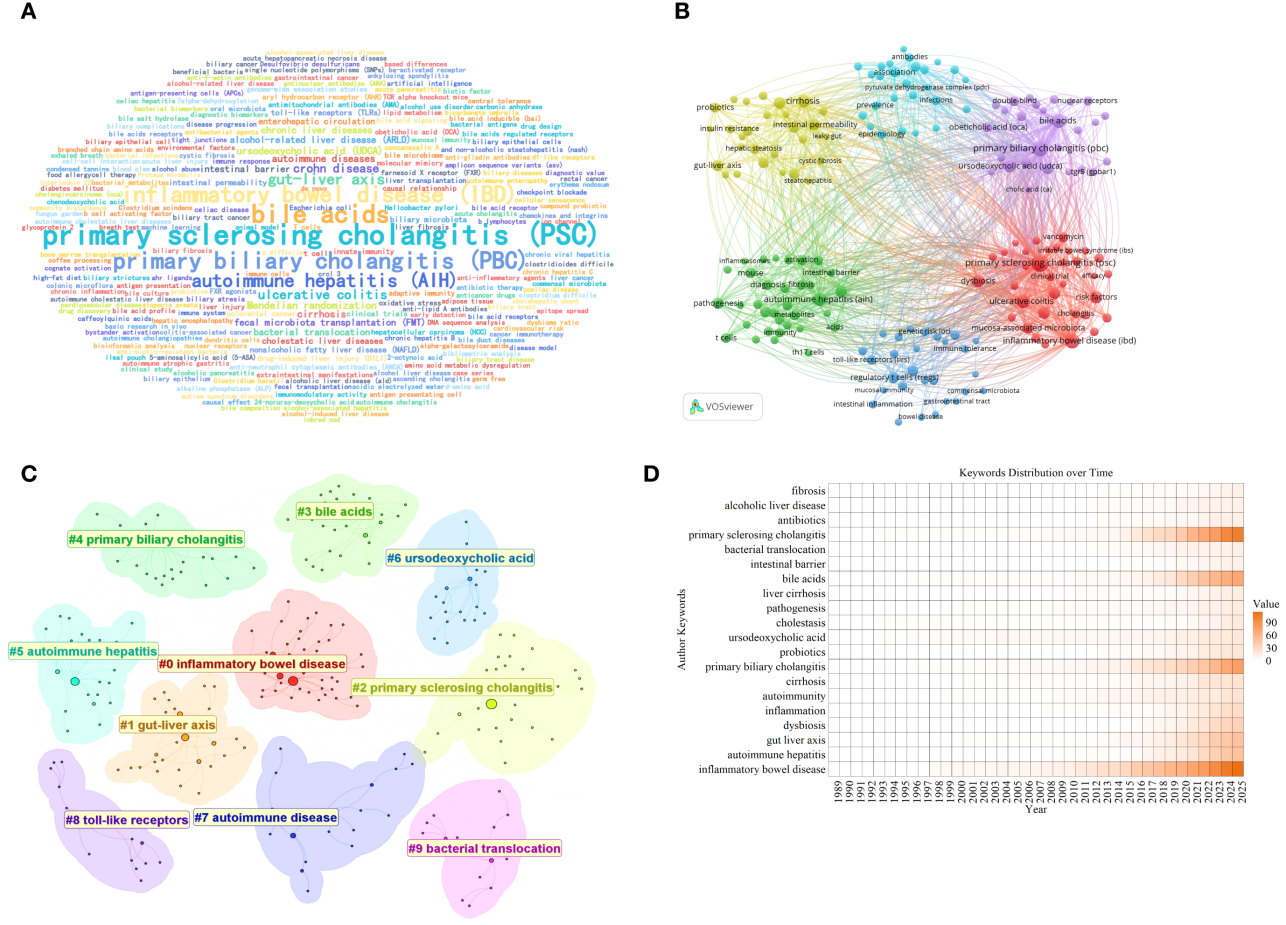
Keywords analysis of research on AILD and gut microbiota. **(A)** Keywords word cloud diagram. Words with larger font sizes indicate higher occurrence frequency and greater research attention. **(B)** Keywords co-occurrence network. Nodes of different colors represent the keywords in different clusters. The size of the nodes indicates total link strength, and the thickness of the lines represents the link strength between two keywords. **(C)** Keywords cluster diagram. Different colors represent different keyword clusters, and the cluster names indicate the themes of each cluster. The smaller the cluster ID, the greater the number of keywords it contains, and the more popular the research within that cluster. **(D)** Heatmap of keywords,distribution over time. Darker colors indicate higher annual occurrence frequency.

Keywords represent the core content of an article, and their visual analysis aids in identifying the latest research dynamics and future development directions within the field. **Figures 8A, B** present the keyword word cloud and co-occurrence network, respectively. Keywords such as “primary sclerosing cholangitis (PSC)”, “primary biliary cholangitis (PBC)”, “inflammatory bowel disease (IBD)”, and “bile acids” appear frequently in the literature with high total link strength, exhibiting a pronounced clustering effect. This reflects the rapidly increasing academic attention on these topics. **Figure 8C** displays ten distinct clusters identified using the k-means clustering algorithm, encompassing: “inflammatory bowel disease”, “gut-liver axis”, “primary sclerosing cholangitis”, “bile acids”, “primary biliary cholangitis”, “autoimmune hepatitis”, “ursodeoxycholic acid”, “autoimmune disease”, “toll-like receptors”, and “bacterial translocation”. The annual heatmap of keywords illustrates their temporal evolution trends (**Figure 8D**). In recent years, the frequency of keywords such as “primary sclerosing cholangitis (PSC)” and “primary biliary cholangitis (PBC)” has surged dramatically, indicating that research into the immune mechanisms of these diseases is gradually becoming a frontier issue in academia. The heatmap also reveals that bile acid metabolism and inflammatory bowel disease have emerged as new themes in this research field in recent years, a trend expected to strengthen further in the coming years.

**Figure 9:**
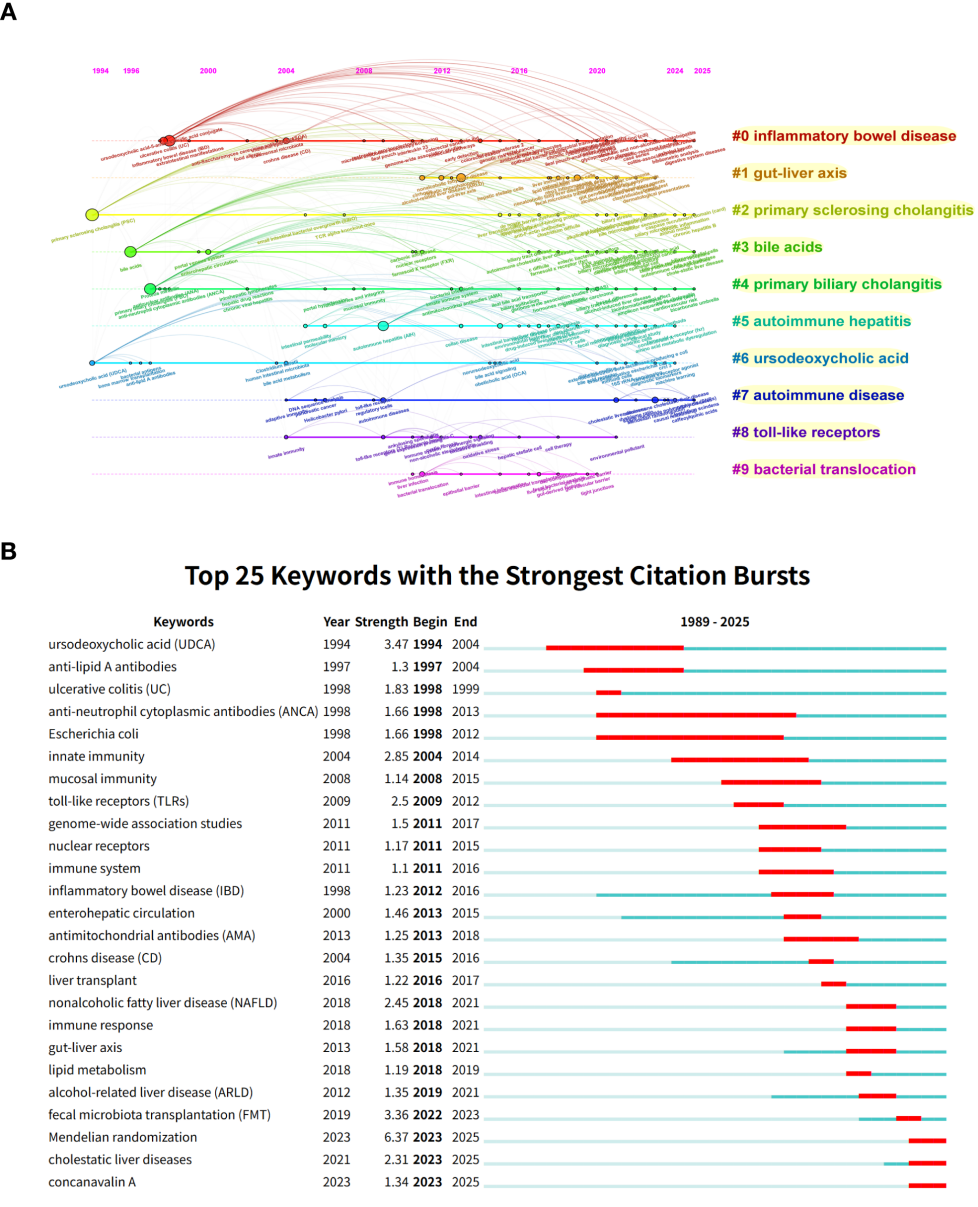
Keywords analysis of research on AILD and gut microbiota. **(A)** Keywords timeline. Each timeline corresponds to a cluster, reflecting the temporal distribution of keywords occurrence from left to right. Circle size indicates keyword frequency, while the curved connection lines represent keywords co-occurrence. **(B)** Top 25 keywords with the strongest citation bursts. The red segment indicates the time span of the keyword’s citation burst.

The keywords timeline generated using CiteSpace software clearly illustrates the evolution of research focus in scientific studies (**Figure 9A**). CiteSpace divides keyword evolution into ten distinct timelines, highlighting recent research hotspots including “inflammatory bowel disease”, “primary sclerosing cholangitis”, “bile acids”, “primary biliary cholangitis”, and “autoimmune disease”. The keywords burst map (**Figure 9B**) indicates that “Mendelian randomization” is the term with the strongest citation burst (strength = 6.37), exhibiting high intensity between 2023 and 2025, followed by “ursodeoxycholic acid (UDCA)” (strength = 3.47). Research focus has progressively shifted toward mechanisms of the gut-liver axis and genetics, evolving from the citation surges of “ursodeoxycholic acid (UDCA)” and “anti-lipid A antibodies” in the 1990s, to the rise of “innate immunity” and “toll-like receptors (TLRs)” in the 2000s, and most recently to the emergence of research methods like “fecal microbiota transplantation (FMT)” and “Mendelian randomization”.

### 3.9 Validation of bibliometric results

**Figure 10:**
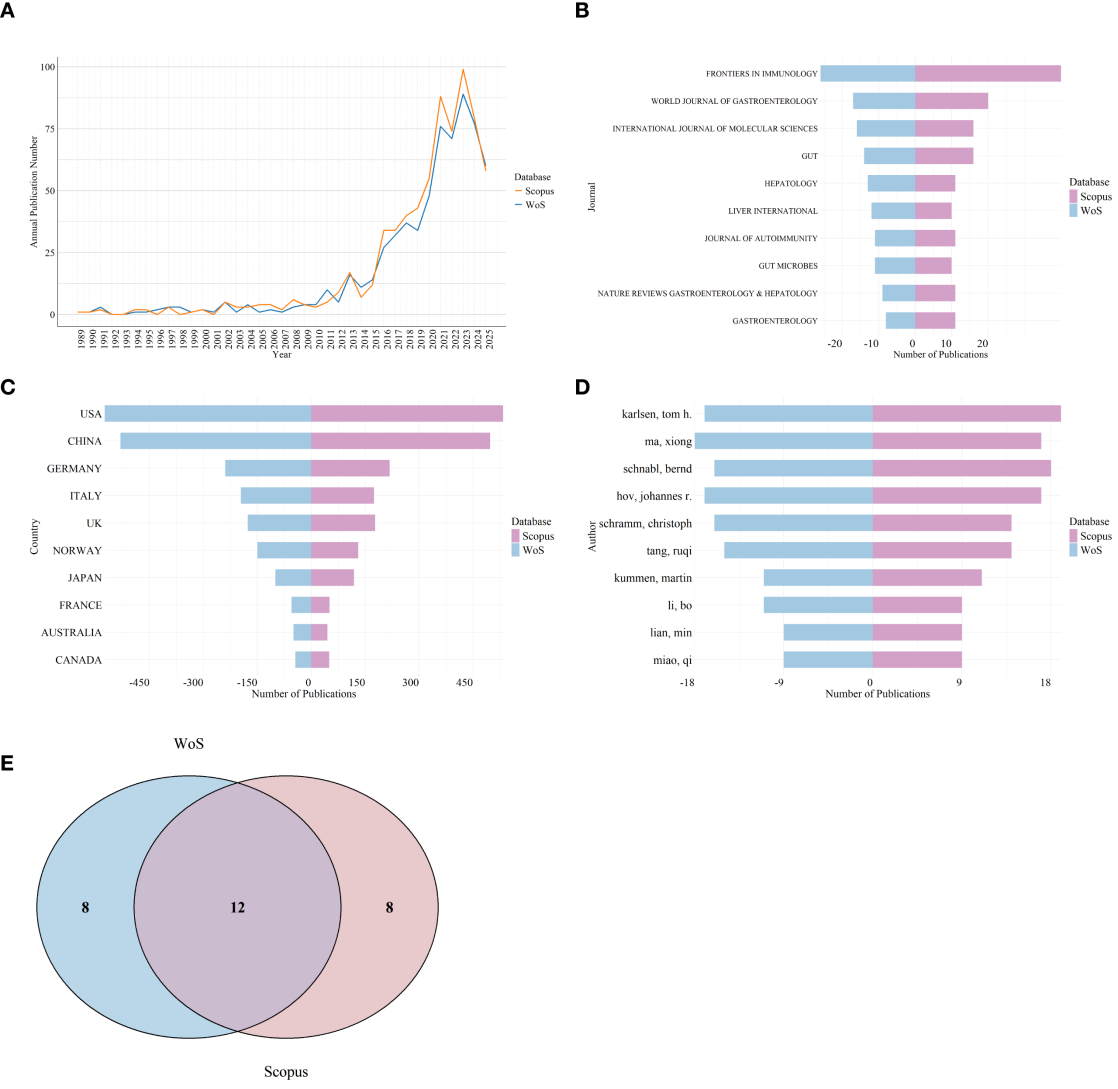
Cross-database validation of bibliometric results using Scopus. **(A)** Annual publication trends comparison between WoS and Scopus. **(B)** Top10 leading contributing journals comparison between WoS and Scopus. **(C)** Top10 leading contributing countries/regions comparison between WoS and Scopus. **(D)** Top10 leading contributing authors comparison between WoS and Scopus. **(E)** Venn diagram showing the overlap of high-frequency keywords in WoS and Scopus.

Although the total number of publications retrieved from Scopus was slightly higher than that from WoS, the overall temporal trends in annual publication output were highly consistent across the two databases (**Figure 10A**). The distributions of leading contributing journals, countries/regions and authors showed similar patterns in both datasets (**Figures 10B–D**). In addition, 60% of top20 high-frequency keywords identified in WoS were also present among the top20 high-frequency keywords in Scopus (**Figure 10E**). These results support the robustness of the main bibliometric findings.

## 4 Discussion

### 4.1 General information

This study collected literature on AILD and gut microbiota from the Web of Science database up to October 13, 2025. Bibliometric analysis and visualization were conducted using tools including VOSviewer, CiteSpace, R, Bibliometrix, SciExplorer, and WPS Office. It quantitatively analyzed fundamental information including annual publication volume, journals, disciplines, countries, institutions, and authors within this field, while reviewing research outcomes and progress. This is the first study to investigate publications in this field using bibliometric analysis methods. We found that the relationship between AILD and the gut microbiota is receiving increasing attention. Especially after 2016, the annual publication volume surged dramatically, coinciding with the global increase in research investment in the microbiome. This phenomenon also reflects the fact that with the advancement of science and technology, especially the rapid development of molecular biology, genomics and high-throughput sequencing technology, as well as the introduction of the concept of the “gut-liver axis”, which allows us to analyze the infinitely small world in the human body that may have a great impact on human health, the link between gut microbiota and AILD is gradually being widely recognized and explored.

The journals Frontiers in Immunology and World Journal of Gastroenterology lead the field in both publication volume and H-index, reflecting the growing evidence of the reciprocal relationship between the gut microbiota and the host immune network. Gershwin M. Eric is a highly productive scholar in the field of AILD and gut microbiota interactions. His team proposed the “bile acids-gut microbiota-cholestasis” triadic relationship (21) and identified compositional and functional alterations in the gut microbiota of AIH patients (10).

The United States leads the world in both the number of publications and citation counts in the fields of AILD and gut microbiota research, a phenomenon driven by multiple factors. On the one hand, the United States holds a significant advantage in terms of policy and strategic planning for advancing AILD and gut microbiome research. In 2007 the National Institutes of Health (NIH) launched the “Human Microbiome Project (HMP)” (22), investing hundreds of millions of dollars to provide continuous funding for research on the relationship between microbiome function and health/disease (23). In 2016, the White House Office of Science and Technology Policy (OSTP) partnered with multiple federal agencies to launch the “National Microbiome Initiative (NMI)” (24). This initiative incorporated microbiome research into the national science strategy, fostering interagency collaboration and resource sharing, and provided long-term policy support for subsequent research on the microbiome and immune-mediated diseases. Additionally, the “NIAID Microbiome Program” at the National Institute of Allergy and Infectious Diseases (NIAID) (25), an agency under the NIH, provides significant support for research on the relationship between microbiota and host immunity. Together, these efforts have propelled the United States to a leading position in the field of gut microbiota and AILD research. On the other hand, the United States possesses advanced scientific research infrastructure, with top academic institutions such as Harvard University and the Mayo Clinic cultivating a large number of interdisciplinary research talents. This enables researchers to access high-quality samples and technologies, driving close collaboration between scientific research and clinical applications.

As shown in **Figures 4D, E** collaboration between Europe/America and China is the most extensive. Transcontinental scientific cooperation effectively bridges gaps in distinct research directions and advances global studies on the interaction between gut microbiota and AILD. Western countries, particularly the United States and European nations such as Germany and the United Kingdom, possess formidable strengths in basic medical research. Meanwhile, China, India, and other Asian countries hold unique advantages in multi-center sample collection and large-scale data studies. Currently, epidemiological data on AILD in South America and Africa is severely lacking, and research on the relationship between AILD and gut microbiota is even scarcer. This phenomenon may be attributed to factors such as insufficient research funding, political and economic instability, and the inaccessibility of advanced medical services. However, the gut microbiota of South American and African populations have been revealed to possess extraordinary taxonomic richness and functional diversity, not fitting the simple “western-nonwestern” axis model (26–32). Their diversity in genetic backgrounds, environmental exposures, and dietary structures presents unique opportunities for research on this cross-cutting theme. In the future, the AILD cohort microbiome database can be established for these research gap areas, and comparative cross-continental analyses can be conducted to explore the influence of genetic, ethnic, geographic, and dietary factors on AILD-gut microbiota.

Although there were slight differences in the number of articles retrieved from Scopus and WoS, annual publication trends, high-frequency keywords, leading contributing journals, leading contributing countries/regions and leading contributing authors were approximately consistent across both databases, confirming the reliability and generalizability of our research findings. The additional articles found in Scopus may be attributed to the broader indexing coverage of Scopus, which includes more journals, especially in emerging fields and regional publications.

### 4.2 Hotspots and frontiers

Keyword analysis can reveal hot topics and research trends in the field. In this bibliometric analysis, “Mendelian randomization” emerged as the keyword with the strongest citation burst, continuing its burst in 2025. This reflects researchers’ urgent need to identify causal relationships between AILD and gut microbiota. “Primary sclerosing cholangitis”, “primary biliary cholangitis”, “bile acids”, and “gut-liver axis” have emerged as key research areas in recent years. Given their inextricable relationship within the field of AILD, we integrate these topics with “Mendelian randomization” for comprehensive discussion.

A growing number of studies have revealed reduced gut microbiota diversity and altered abundance of specific microbial communities in AILD patients, indicating that the gut microbiota is associated with disease progression and treatment response in AILD (10, 11, 33–37). This has positioned microbiota-targeted interventions as a potential novel avenue for clinical diagnosis and treatment. confounding variables such as environmental factors, lifestyle, and age. It remains difficult to determine whether alterations in the gut microbiota are causative or driving factors of AILD, or merely side effects or changes occurring during the clinical course triggered by the medical interventions or disease itself. MR serves as the key research method to overcome this challenge. As an epidemiological approach that relies on genetic variation as an instrumental variable to infer causal relationships between exposure and outcome, MR effectively controls for confounding factors and reverse causality issues (38). Its methodological advantages have been recognized by most researchers, demonstrating its ability to reduce confounding and misinterpretation risks, thereby moving closer to causal inference rather than mere correlation analysis.

The application of MR in the field of AILD-gut microbiota interactions has gradually shifted from theoretical exploration to empirical investigation. Cui et al. identified through MR that the relative abundances of the genus *Butyricicoccus*, the genus *Erysipelatoclostridium*, and the genus *Clostridium innocuum* group were all negatively correlated with PBC risk; the relative abundance of the genus *Eubacterium hallii* was positively correlated with PSC risk, while the relative abundances of the family *Lachnospiraceae* and the family *Clostridiaceae* 1 were both negatively correlated with PSC risk (39). Previous studies have also found that *Clostridium subcluster XIVa* is reduced in PBC patients compared to the control group (40). Patients with PBC usually experience disorders in bile acid metabolism and weakened dehydroxylation reactions, which leads to an increase in the concentration of hydrophobic primary bile acids, thereby causing liver and bile duct cells damage and toxic accumulation (41, 42). In the colon, nearly all bile acids undergo biotransformation into secondary bile acids via 7α-dehydroxylation. However, only approximately 0.0001% of colonic bacteria are capable of performing this reaction, including *Clostridium* (clusters XIVa and XI) and *Eubacterium* of the *Firmicutes* phylum (43). Staley et al. and Furukawa et al. also confirmed that Clostridium can convert primary bile acids into secondary bile acids via 7α-dehydrogenation (11, 44). Therefore, *Clostridium* may reduce the risk of developing PBC through this mechanism. Additionally, *Clostridia* is one of the dominant commensal bacteria capable of inducing the production of colonic regulatory T (Treg) cells, which play a central role in suppressing inflammation and allergic reactions (45, 46).

Besides, MR analysis conducted by Fu et al. (16) demonstrated that the genus *Clostridium innocuum* group also exhibits significant protective effects against AIH. Moreover, its belonging order *Clostridiales* has been utilized as a biomarker to distinguish AIH from control groups in microbial diagnostic models (10). However, the study also indicates that *Ruminiclostridium* 9 exhibits a protective effect in AIH, while the presence of the *Ruminiclostridium* 5 is associated with an increased risk of PBC. Given that some patients may simultaneously exhibit AIH-PBC overlap syndrome (47), or even AIH-PBC-PSC overlap (48), caution should be exercised when interpreting these results. Further research is needed to elucidate the role of these microbial characteristics in the pathogenesis of AILD.

More specific MR empirical results are also beginning to emerge. Zhang et al. (15) pioneered the use of immune cells as mediators, revealing the causal relationship between the gut microbiota and AILD while elucidating the underlying regulatory mechanisms of immune cells. Through MR analysis, they discovered that the *species Ruminococcus obeum* genetically downregulates CD62L-CD86+ myeloid Dendritic Cell %Dendritic Cells(DC), with this pathway partially mediating its protective effect on PSC. This finding provides a novel perspective for future therapeutic targets in PSC. Research by Lapidot et al. also found that the abundance of *Ruminococcus* in the gut microbiota of PSC patients was reduced (49). Short-chain fatty acids (SCFAs), including acetic acid and butyric acid, are the end products of dietary fiber fermentation by gut microbiota, which maintain the integrity of the intestinal mucosa through multiple pathways (50–53). *Ruminococcu* are important SCFAs-producing bacteria in the human gut. A reduction in their abundance decreases SCFAs production and compromises the intestinal barrier (54). As sentinel antigen-presenting cells, DCs are located near or integrated into epithelial cell interfaces at the boundary between our bodies and the external environment (such as the lungs and intestines). During antigen acquisition, DCs exposed to inflammatory signals upregulate the expression of the co-stimulatory molecule CD86 and lymph node homing chemokine receptors. They activate helper T cells (e.g., Th1, Th17) after migrating to lymph nodes (55, 56). In summary, we hypothesize that in susceptible individuals, the absence of the *species Ruminococcus obeum* leads to intestinal mucosal inflammation. Massively activated CD62L-CD86+ DCs abnormally migrate to the bile duct surrounding area, where they abnormally activate pathogenic T cells targeting bile duct epithelial antigens. Concurrently, intestinal inflammation induces abnormal expression of gut-specific adhesion molecules (e.g., MAdCAM-1) on bile ducts, promoting abnormal homing of helper T cells expressing corresponding homing receptors (e.g., α4β7 integrin) to the bile ducts. This synergistically drives the development of PSC.

Zhou et al. (57) revealed through bidirectional MR analysis that PBC may not only be influenced by gut microbiota (order *Coriobacteriales* and class *Deltaproteobacteria*), but also that the disease progression of PBC reciprocally affects the abundance of gut microbiota (class *Bacteroidia*, order *Bacteroidales*, phylum *Bacteroidetes* and genus *Lachnospiraceae* UCG010). This bidirectional causal model not only reveals the complex relationship of the gut-liver axis but also provides new insights for microbiome intervention strategies in AILD. This study indicates that class *Deltaproteobacteria* may provide protective effects against PBC. The Family *Desulfovibrionaceae* is a subfamily within the class *Deltaproteobacteria*. A review study indicated that the abundance of the genus *Desulfovibrio* was lower in patients with PBC compared to healthy controls (58).

In summary, MR studies partially overcome confounding and reverse causality issues by utilizing genetic variants associated with specific microbial traits as instrumental variables, thereby providing more reliable evidence for assessing the potential causal influence of gut microbiota on AILD. However, the more exciting direction lies in microbiome intervention. Beneficial strains or metabolites identified through MR and mechanism studies hold promise for becoming the next generation of probiotics for treating AILD. The most promising approach is not a “miraculous strain”, but a set of microecological regulation strategies targeting the key nodes of the “gut-liver axis”. Among them, the microbiota aimed at restoring immune tolerance (e.g., butyrate-producing bacteria) and repairing the gut-liver barrier (e.g., Akkermansia muciniphila) show the greatest potential.

*Clostridium butyricum* is a butyric acid-producing, spore-forming, Gram-positive anaerobic bacterium. In 1933, Dr. Chikaji Miyairi first isolated *Clostridium butyricum* from the feces of healthy individuals. In 1963, *Clostridium butyricum* MIYAIRI 588 (CBM 588), isolated from soil, was developed into a pharmaceutical and widely used in Japan to treat gastrointestinal disorders such as diarrhea (59). Among various possible mechanisms, butyrate is commonly regarded as the primary reason for CBM 588’s efficacy. Multiple studies indicate that CBM 588 positively influences gut microbiota composition, short-chain fatty acid levels (such as butyrate), and intestinal barrier function, while also regulating host immune-metabolic networks (60–62). Animal studies indicate that CBM588 demonstrates positive effects in models of non-alcoholic fatty liver disease, colitis, ulcerative colitis (UC), and irritable bowel syndrome (IBS) (63–67). CBM588 has also demonstrated potential applications in human gastrointestinal diseases, psychiatric disorders, neurological conditions, and metabolic diseases (62). However, there is currently no clinical or basic research evidence supporting the direct use of CBM 588 for treating AILD. Further mechanism-based human studies are required to determine the clinical application value of CBM 588 in AILD.

### 4.3 Limitations

This study has certain limitations. First, due to software limitations, the analysis only included literature from the WoSCC database, potentially omitting literature from other databases. However, as this database is the most reliable global repository for scientific and academic research, and we selected literature from the Scopus database for verification, our findings remain credible. Second, this study only included English-language literature, which may introduce bias in the results. Finally, the use of different combinations of literature analysis software may result in subtle variations in findings. Despite these limitations, our study provides a comprehensive analysis of hotspots and trends in the AILD-gut microbiota field, offering important clues for current and future research efforts.

## 5 Conclusions

This study reveals the current research status and development trends of gut microbiota in the field of AILD. By integrating MR, it explores the crucial role gut microbiota plays in the pathogenesis of AILD, particularly through immune regulation driven by the gut-liver axis and microbial metabolites. Although current research has made some progress, many unresolved questions remain regarding the clinical application of gut microbiota. Future research should expand the global microbiome database for AILD cohorts, integrating factors such as genetics, ethnicity, geography, and diet to further clarify the therapeutic potential of gut microbiota in AILD.

## Data Availability

The raw data supporting the conclusions of this article will be made available by the authors, without undue reservation.
